# A Critical Review on Desalination Technologies for High-Salinity Wastewater: Development and Challenges

**DOI:** 10.3390/membranes16010027

**Published:** 2026-01-03

**Authors:** Xiao Wang, Xinyi Cheng, Ao Shuai, Xiyu Xu, Xinran Guo, Dan Song, Caihong Liu, Wenjuan Zhang

**Affiliations:** 1 Key Laboratory of Eco-Environments in Three Gorges Reservoir Region, Ministry of Education, College of Environment and Ecology, Chongqing University, Chongqing 400044, China; 13284606099@163.com (X.W.); 18716241890@163.com (X.C.); shuaiao001@gmail.com (A.S.); 15090492612@163.com (X.X.); 17823374028@126.com (X.G.); 2 State Key Laboratory of Urban Water Resource and Environment, Harbin Institute of Technology, Harbin 150090, China; songd@hit.edu.cn; 3 Tianjin Key Laboratory of Aquatic Science and Technology, School of Environmental and Municipal Engineering, Tianjin Chengjian University, Tianjin 300384, China

**Keywords:** high-salinity brine, membrane-based desalination, thermal desalination, biological desalination, zero liquid discharge

## Abstract

The ongoing expansion of industrial operations has resulted in the generation of a large amount of high-salinity wastewater with complex compositions. The direct discharge of this wastewater poses significant threats to ecosystems and leads to the loss of valuable salt resources, for example, triggering freshwater salinization syndrome and mobilizing heavy metals to form toxic “chemical cocktails”, leading to the loss of valuable salt resources. Desalination of high-salinity wastewater primarily involves two key processes: concentration and crystallization, whereby a concentrated brine is first obtained through membrane-based or thermal methods, followed by salt recovery via crystallization. This review begins by employing a bibliometric analysis to map the knowledge structure and trace the evolution of research trends, revealing that “membrane-thermal integration” has become a dominant research hotspot since 2020. It then provides a systematic examination of advanced treatment technologies, chronicling the progression from early biological methods to contemporary membrane-based and thermal desalination approaches. A specific analysis of the influence of salinity on membrane scaling is also included. Consequently, this paper critically assesses the prospects and challenges of several alternative desalination technologies and proposes that integrated processes, combining membrane-based and thermal desalination, represent a highly promising pathway for achieving zero liquid discharge (ZLD). Finally, we suggest that future research should prioritize the development of key functional materials, explore efficient hybrid physiochemical–biochemical processes, and advance emerging technologies, aimed at enhancing treatment efficiency and reducing operational costs.

## 1. Introduction

As the global population continues to grow, water scarcity has become an increasingly pressing issue in many regions. In particular, the availability of fresh water is severely limited in areas with high salinity, such as coastal regions and arid regions [1]. Freshwater accounts for merely 3% of Earth’s total water, and excessive withdrawal of natural reserves, inefficient management, along with climate change and pollution, are expected to expose 4 billion people to global water stress by 2030 [2]. High salinity water refers to water containing a high concentration of dissolved solids [3]. Significant volumes of complex high-salinity wastewater are generated by various industrial activities, including coal chemical processing, textile manufacturing, desalination of seawater and shale gas extraction [4,5,6]. In 2022, China treated 30.16 billion tons of industrial wastewater. Assuming that brine wastewater accounts for >5% of the total production and has an estimated salt content of 1%, the annual production of by-product salt is approximately 1.5 × 10^7^ t [7]. Among these, shale gas extraction produces shale oil and gas wastewater (SOGW) with total dissolved solids (TDS) up to 300,000 mg/L—far exceeding seawater’s ~35,000 mg/L. This wastewater also contains fracturing additives, heavy metals, and radioactive substances, presenting considerable treatment difficulties [8]. Discharging high-salinity water directly into freshwater bodies can lead to freshwater mineralization, organic-induced eutrophication, and the accumulation of heavy metals in the food chain, all of which pose serious risks to ecosystems [9,10]. Therefore, developing rapid and efficient treatment technologies for high-salinity wastewater has become a vital focus in the field of environmental water management.

As water treatment technologies advance, a growing number of methods for treating high-salinity water have been developed, accompanied by a substantial increase in related research publications [11]. However, conventional treatment approaches often prove either ineffective or economically prohibitive for high-salinity contexts [9]. For instance, atmospheric water harvesting (AWH)—an emerging decentralized technology—remains more energy-intensive than conventional water treatment accounting for 84% of the global population, restricting its large-scale application [12]. The increasing emphasis on zero liquid discharge (ZLD) in industrial wastewater management has further stimulated technological innovations in high-salinity wastewater treatment [13]. As a result, alternative strategies such as membrane-based desalination, thermal desalination, and other emerging methods have been developed to improve the efficiency and feasibility of treating high-salinity water [11].

While a growing number of studies have been investigated on high-salinity wastewater treatment in recent years, most studies remain confined to specific saline water sources or a narrow technological scope—such as focusing solely on membrane-based or biological methods. Moreover, discussions often highlight technological limitations in desalination performance, and explorations of ZLD tend to address only isolated aspects of a single technology. These constraints lead to an incomplete and often fragmented understanding of the field. To address these gaps, this review aims to provide a comprehensive and critical analysis of the current state of high-salinity wastewater treatment technologies. Our primary objective is to synthesize contributions from various treatment processes reported over the past three decades. We present a systematic overview of commonly used technologies, outline their fundamental working principles, and summarize recent research progress. Furthermore, we conduct a comparative assessment of the advantages and limitations of each technology, identify key challenges and future research priorities, and emphasize the importance of zero-liquid-emission strategies as a forward-looking direction. The review concludes by discussing remaining obstacles and offering a perspective on the future of high-salinity water desalination.

## 2. Bibliometric Analysis of Literature Characteristics

### 2.1. Spatial and Temporal Trends in Published Documents

**Figure 1:**
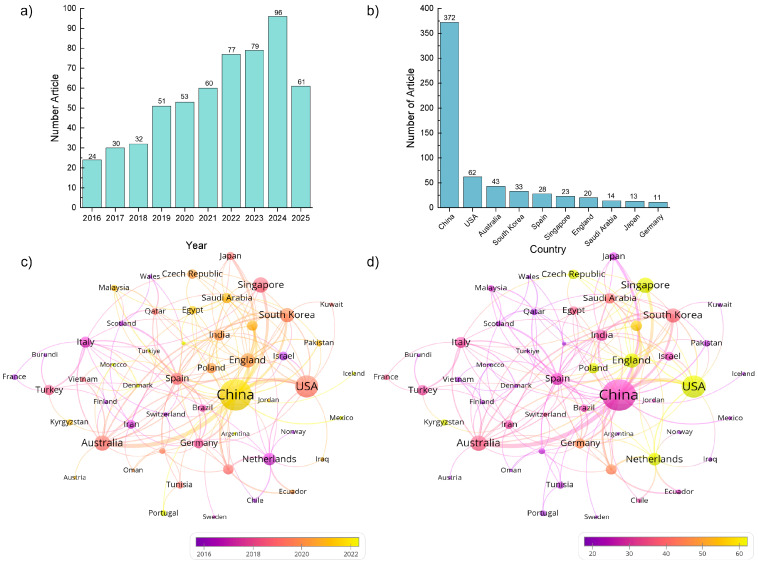
**Visual analysis of publication year, country.** (**a**) Annual documents, (**b**) top 10 contributed countries and (**c**) The average publication year (**d**) and average citation of documents published by different countries.

We retrieved literature from the Web of Science Core Collection (WoSCC), and the language was limited to English. VOS viewer 1.6.20 software was used to analysis the countries, journal sources, and keywords. Data organization was performed by Microsoft Excel 2016, and related figures were drawn with Origin 2022 software. The analyzed documents consisted of a total of 656 documents, which were published from 1995 to 2025. Figure 1a specifically presents the data from 2016 to 2025. Prior to 2016, only 98 documents related to high salinity wastewater treatment were recorded. However, in the following years, there was a significant surge in documents, with a total of 582 documents published between 2016 and 2025, accounting for 85.06% of the total. Notably, the highest number of documents occurred in 2023 and 2024, with 96 documents, taking up 14.634% of all documents. This trend signifies the growing interest of researchers in the field of high salinity wastewater treatment. Figure 1b illustrates the global distribution of research on high salinity wastewater treatment by country. Among the 656 analyzed documents, China emerged as the leading contributor with 372 documents, followed by the United States with 62 documents. Other notable contributors include Australia (43 documents), South Korea (33 documents), and Spain (28 documents). The fact that research on high salinity wastewater treatment has been conducted in 59 countries reflects the widespread concern and engagement in addressing this issue globally.

Figure 1c shows a network visualization of international cooperation in high-salinity water treatment research, where the size of the circles represents the number of published documents, the lines indicate the level of cooperation between countries, and the color indicates the average year of publication. The color reveals that the United States, Germany, and the United Kingdom tended to publish documents on high salinity wastewater treatment earlier, while China was a latecomer. This suggests that these countries played an important role in early research on high-salinity wastewater treatment, while China’s research has rapidly grown in recent years. Figure 1d reveals that China, despite having the highest number of published documents, does not demonstrate outstanding performance with an average citation count of 26.70. The United States, being the second highest in terms of published documents, also ranks second in terms of average citation count with 68.53. The country with the highest average citation count is Netherlands, which published 13 documents with an average citation count of 71.46.

### 2.2. Analysis of Main Keywords

#### 2.2.1. Temporal Trend of Keywords

**Figure 2:**
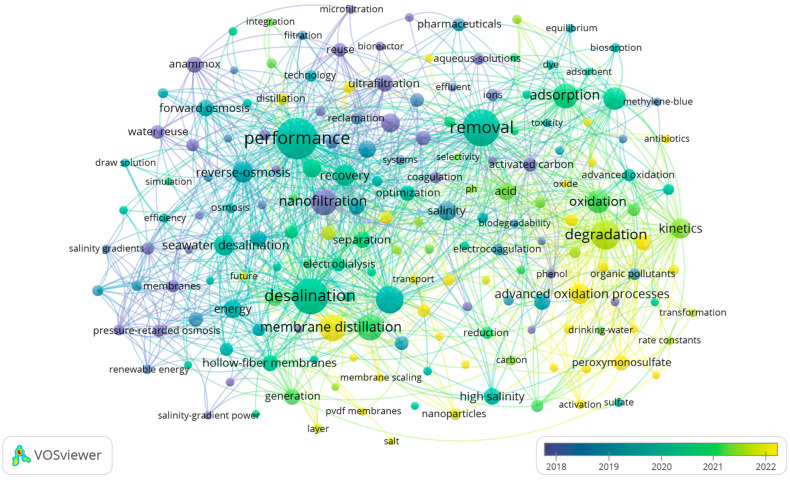
Temporal distribution network of keywords about high salinity wastewater treatment.

Figure 2 displays the average year of keywords occurrence based on an analysis of 656 publications on high salinity water treatment. The purple keywords, such as “anammox” and “membrane bioreactor”, indicate that early research efforts primarily centered on these two areas. Regarding biological treatment, other keywords associated terms like “microbial community”, “aerobic granular sludge”, “salt-tolerant bacteria”, and “activated sludge” suggest that biological treatment of high salinity water is highly dependent on microbial and sludge cultivation. A detailed discussion of biological treatment techniques for high-salinity water follows in the subsequent section.

With the development of research on high salinity water treatment, new keywords such as “forward osmosis”, “membrane scaling”, “membrane bioreactor”, “membrane distillation”, and “desalination” have emerged. This trend reflects a growing recognition and application of membrane-based technologies in this field. Concurrently, the emergence of terms like “solar evaporation” signifies the parallel development and application of thermal approaches for treating high-salinity water.

Recent research trends are reflected in keywords such as “zero liquid discharge”, “membrane scaling”, “electrochemical oxidation”, “solar evaporation”, and “singlet oxygen”. ZLD aims for complete liquid waste elimination by recovering all water from the feed stream, leaving only solid residues—a goal increasingly driven by stringent environmental regulations. In membrane technology development, challenges such as membrane fouling and membrane scaling remain critical obstacles. Meanwhile, to address energy consumption concerns, studies on solar evaporation have emerged, which largely rely on solar energy and often incorporate advanced materials like graphene oxide. Additionally, advanced oxidation processes like electrochemical oxidation and the involvement of reactive species such as singlet oxygen highlight innovations in pollutant removal strategies.

#### 2.2.2. Keywords Cluster Analysis

**Figure 3:**
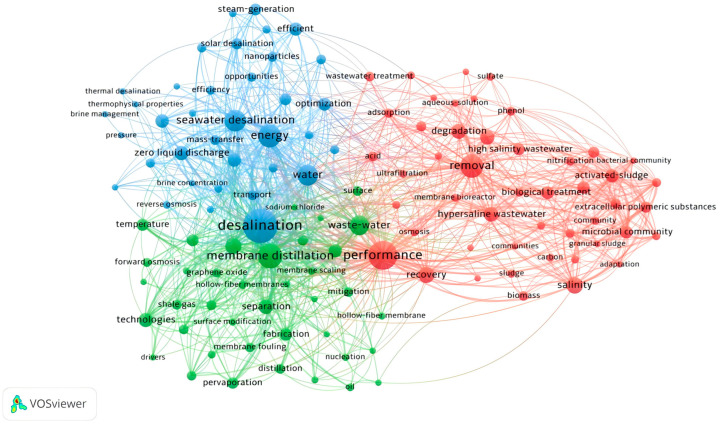
Cluster analysis network of keywords about high salinity wastewater treatment.

**Figure 4:**
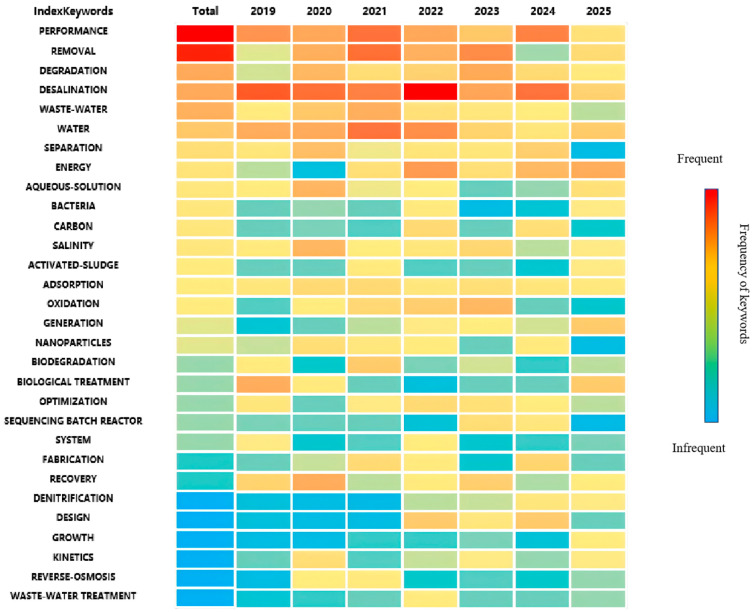
Index keywords of the research of high-salt wastewater.

Figure 3 categorizes the keywords into three clusters: red, blue, and green. The red cluster contains microbiology-related terms such as “biological treatment”, “microbial community”, and “activated sludge”, indicating the significant role of biological processes in treating high-salinity water. When correlated with Figure 4, it becomes evident that biological treatment was a major research focus in the field’s early stages. Keywords in the green cluster, including “membrane distillation,” “forward osmosis,” and “membrane scaling,” identify it as being centered on membrane-based desalination. The repeated emphasis on “membrane scaling” highlights that it is a primary technical obstacle and a key focus of research in this domain.

## 3. Hot Spots and Future Research Needs

The desalination of high-salinity water has numerous practical applications. As previously analyzed, the relevant technologies can be categorized into three primary areas: membrane-based desalination, thermal desalination, and biological treatment. An analysis of research trends in Figure 4 reveals the evolving focus within this field. While the treatment capacity and removal efficiency of technologies have remained consistent research hotspots, the problem of energy consumption is receiving increasing attention. Notably, the focus on biological treatment has declined in recent years, while membrane-based desalination has emerged as a prominent development trend. Furthermore, ZLD is frequently highlighted in the literature as a critical future direction for the development of high-salinity desalination technology. The following sections will introduce and compare the three main technological approaches, explain the critical influence of salinity on membrane scaling, and discuss the guiding significance of ZLD for future research.

### 3.1. Desalination Technology for High-Salinity Wastewater

#### 3.1.1. Biological Desalination

Biological treatment has long been a cornerstone of wastewater treatment plants; however, its application in high-salinity wastewater is significantly constrained by the adverse effects of salt on microbial activity. High salt levels can compromise cell membrane integrity and permeability, impairing the uptake and utilization of organic substrates. Additionally, elevated salinity disrupts enzymatic functions and metabolic pathways, leading to inhibited degradation and conversion of organic pollutants [14]. To overcome these limitations, various biological strategies have been developed, including aerobic and anaerobic processes, as well as the cultivation and acclimation of salt-tolerant or halophilic microorganisms.

Aerobic granular sludge (AGS), with its dense structure and high settling velocity, offers a more compact alternative to conventional activated sludge, particularly under saline conditions [15]. For instance, one study reported over 95% removal of BOD, COD, ammonia nitrogen, and total phosphorus in a sequencing batch reactor (SBR) operating at 2.0 wt% salinity using acclimated activated sludge [16]. Anaerobic processes are also gaining attention due to their low energy requirements. The discovery of anaerobic ammonium oxidation (anammox) in marine environments suggests its potential applicability in saline wastewater treatment [17,18]. In a study on fish canning wastewater, introducing an anaerobic feeding phase improved organic removal efficiency to 80–90%, outperforming a fully aerobic system (75–85%) [19].

Another promising approach involves the use of salt-tolerant or halophilic microorganisms [20,21]. While salt-tolerant species can survive at high salinity without relying on salt for growth, halophiles require saline conditions [22]. These organisms maintain osmotic balance through the accumulation of compatible solutes or by adjusting membrane ion composition, and their enzymes often remain active and stable under high-salt conditions [23]. The development and domestication of such strains represent an active research area.

More recently, microbial desalination cells (MDCs) have emerged as an innovative technology that combines bioenergy production with desalination. In MDCs, exoelectrogenic bacteria in the anode oxidize organic matter, generating a potential gradient that drives ion migration through ion-exchange membranes, thereby desalinating water while treating wastewater [24]. This system has been successfully applied to high-salinity wastewaters, such as mustard tuber wastewater, achieving concurrent COD removal and desalination. Coupling MDCs with reverse osmosis (RO) further reduces energy consumption compared to conventional desalination, highlighting the potential of integrated biological–physical systems for sustainable high-salinity wastewater management.

#### 3.1.2. Thermal Desalination

**Figure 5:**
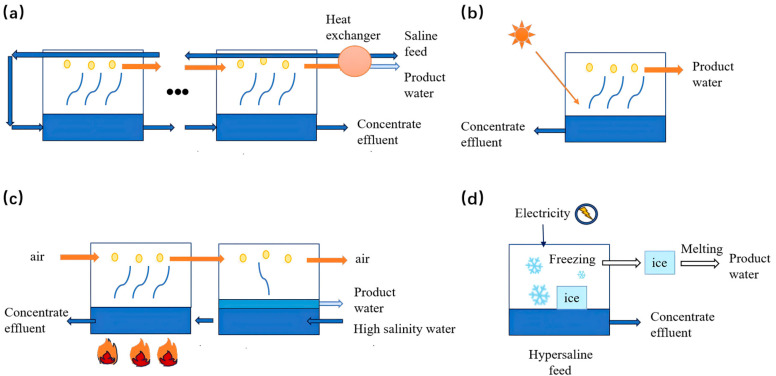
Schematic diagram of (**a**) humidification–dehumidification (HDH) (**b**) solar–thermal desalination (STD), (**c**) multiple effect distillation (MED) and multistage flash (MSF) distillation (ellipses represent repeating units) and (**d**) freeze desalination (FD).

Thermal desalination is a process that removes salts and other impurities from water through evaporation and condensation [25]. A notable advantage of thermal desalination is its ability to utilize waste heat or renewable energy sources—such as solar or geothermal—which helps reduce both energy costs and carbon footprint [26,27]. Although generally less efficient and more costly than membrane-based desalination methods, thermal processes remain advantageous in specific contexts, particularly for treating high-salinity water or in locations with abundant renewable energy resources [11]. Multi-Stage Flash (MSF) and Multi-Effect Distillation (MED) are typical thermal technologies with operating temperatures of 90–110 °C and 70 °C, respectively. MED has a lower electrical energy consumption (1.5–2.5 kWh/m^3^) and CO_2_ emissions (19.2 Kg CO_2_/m^3^) than MSF, making it more competitive when coupled with renewable energy [28]. Among thermal desalination technologies, Humidification–Dehumidification (HDH) employs evaporation and condensation principles to produce fresh water from saline sources [29,30]. As illustrated in Figure 5a, the process involves heating saline water to generate vapor, leaving dissolved salts behind. The vapor is then condensed into fresh water [30].

Solar–thermal desalination (STD) utilizes solar energy to drive thermal desalination processes, often through multiple-effect distillation or membrane distillation [31,32]. As shown in Figure 5b, solar radiation is harnessed to generate steam, which in turn evaporates saltwater to yield freshwater. This approach is particularly suitable for sun-rich regions with limited freshwater access. STD offers a sustainable, renewable alternative that operates without fossil fuels and typically incurs lower operating costs than conventional thermal systems [33]. However, STD also faces challenges, including dependence on consistent solar irradiation, performance variability under adverse weather conditions, high initial investment, and limitations in scalability for large-scale applications [32]. Recent advances in STD have focused on material innovations and structural designs to mitigate salt accumulation and enhance performance. Examples include: Super salt-resistant evaporators with inclined brine regulation for stable operation under high salinity [34]; Waterwheel-inspired rotating evaporators that achieve high evaporation rates (2.8 kg·m^−2^·h^−1^) in saturated brine via self-rotation-induced salt removal [35]; Gradient graphene spiral sponges that enable directional salt crystallization toward ZLD [36]; Ceramic-carbon Janus membranes with hetero-structure designs that attain 66.8–68.8% solar–thermal efficiency, sustaining a flux of 3.3 L·m^−2^·h^−1^ in 90 g/L brine with 99.9% salt rejection [37].

MED and MSF are two established thermal desalination technologies commonly employed for high-salinity water treatment [38,39]. In MED, saline water is heated and passed through a series of evaporator chambers (effects) maintained at progressively lower pressures. Water evaporates in each effect, and the vapor is condensed to produce fresh water, while the remaining brine becomes more concentrated as it moves through the system. This multi-effect arrangement enhances thermal efficiency [38]. In MSF, feed water is heated under high pressure and then introduced into a sequence of flash chambers where the pressure is successively reduced. This causes rapid vaporization (“flashing”) of a portion of the water, and the resulting steam is condensed into fresh water [39]. A major operational challenge for both MED and MSF in high-salinity applications is scaling and corrosion. The precipitation of sparingly soluble salts on heat transfer surfaces and within flow passages leads to fouling, which impairs thermal efficiency and increases pressure losses.

Freeze Desalination (FD) is a process in which saline water is partially frozen to form ice crystals that exclude salts. These crystals are then mechanically separated from the residual brine, yielding fresh water upon melting [40]. FD benefits from low energy requirements and minimal chemical use, but its practical application is limited by low throughput and scaling difficulties [41]. Clathrate Hydrate Desalination utilizes guest molecules (e.g., methane, CO_2_) that form crystalline hydrates with water molecules under specific pressure-temperature conditions, effectively excluding salts. The hydrates are then separated and dissociated to release fresh water. This approach can be driven by low-grade waste heat, though it remains largely at the experimental stage [42].

Emerging thermal desalination approaches also show promise. Thermal Redox Desalination (TRD) leverages temperature gradients to drive reversible redox reactions in ferri/ferrocyanide electrolytes. With a 60 K temperature difference, TRD systems have demonstrated simultaneous salt removal (9.77 μg·cm^−2^·min^−1^) and power generation (188.8 mW·m^−2^), enabling integrated waste heat recovery, desalination, and energy production [43]. All-liquid thermal desalination based on thermodiffusion (Soret effect) uses optimized multichannel configurations to reduce salinity from 30,000 ppm to below 500 ppm through cyclic operation, without phase change or membranes [44].

Other thermal concentration and crystallization technologies include Brine Concentrators (BCo), Brine Crystallizers (BCr), and Solvent Extraction Desalination (SED). Brine concentrators remove water via thermal evaporation and condensation, producing a highly concentrated brine stream. They typically employ multi-effect evaporators to improve energy efficiency [45]. Brine crystallizers further concentrate brines by cooling them below the saturation point of dissolved salts, inducing crystallization. The solid crystals can then be separated for disposal or resource recovery [46]. SED uses selective organic solvents to extract salt ions from saline water. After contact, the solvent phase containing the salts is separated and regenerated by evaporation, yielding both fresh water and solid salt [47,48].

#### 3.1.3. Membrane-Based Desalination

Compared with traditional chemical treatment methods, membrane technology can efficiently remove impurities and harmful substances from high-salinity water [49]. The main technologies include RO, electrodialysis (ED), forward osmosis (FO), and membrane distillation (MD). However, treating high-salinity wastewater is technically challenging due to its complex composition, high salt content, strong inhibition of microbial growth and current RO membranes can typically operate at a maximum pressure of 1000 or 1200 psi (75.8 or 82.7 bar) for seawater desalination RO (SWRO); however, at concentrations higher than SWRO brine (∼70 g/L total dissolved solids (TDS)), the hydraulic pressure required to overcome the osmotic pressure of the brine while achieving practical/economical water production (flux) is above the pressures limits of most membranes manufacturers [50]. These factors make membrane processes susceptible to scaling, which is caused by high concentrations of dissolved solids and organic matter, leading to reduced membrane permeability and lifespan [51,52]. Scaling must be mitigated through measures such as pre-filtration and regular membrane cleaning. Furthermore, the sweep with zero TDS has a recovery of 54% for a feed TDS of 35 g/L, which matches the expected performance of RO processes. As the feed concentration increases, the recovery rate from the RO process drops sharply to only 4% for a feed TDS of 75 g/L [53]. Processes like reverse osmosis require high-pressure drives, resulting in significant energy consumption [54], necessitating optimized system design and operation. The following sections provide an overview of several key membrane technologies used for high-salinity water.

**Figure 6:**
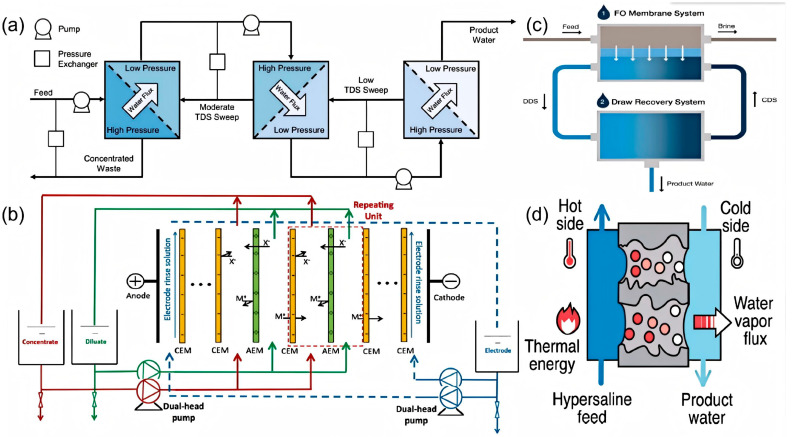
Schematic diagram of the main technology of Membrane-based desalination: (**a**) osmotically assisted reverse osmosis (RO) [53], (**b**) Electrodialysis (ED) [89], (**c**) forward osmosis (FO) [73], and (**d**) Membrane distillation (MD) [11].

RO has become the most prevalent technology for desalinating seawater and brackish water, largely replacing thermal processes in recent decades due to its superior energy efficiency [55,56]. The process operates by using an electrically powered pump to pressurize saline feed water above its osmotic pressure, forcing water molecules through a semipermeable polymeric membrane while rejecting salts and other solutes to produce clean water. RO operates at an electrical energy consumption of 5–9 kWh/m^3^, with CO_2_ emissions up to 8.6 Kg CO_2_/m^3^ and product water TDS < 500 ppm [28]. Despite its effectiveness, RO faces significant challenges in high-salinity applications, primarily membrane scaling, high energy consumption and irreversible membrane compaction. Scaling occurs when high concentrations of salts and dissolved solids clog membrane pores, reducing permeability and increasing operational costs [57,58]. The high pressure required to overcome osmotic pressure also leads to substantial energy consumption [49]. Membrane deformation under an applied hydraulic pressure, often termed compaction, is observed in almost all pressure-driven membrane processes. Most notably, compaction decreases water permeability in conventional RO and is expected to critically hinder high-pressure reverse osmosis (HPRO) for hypersaline brine desalination [59,60]. To further mitigate these drawbacks, researchers are pursuing innovative membrane materials and derivative technologies. For example, graphene oxide membranes intercalated with ionic liquids leverage a “cation-recognition” effect within tailored 2D nanochannels. This design achieves a high water flux of ~32.9 L m^−2^ h^−1^ bar^−1^ while maintaining ~81.4% Na_2_SO_4_ rejection, offering promise for hypersaline treatment [61]. Similarly, pressure-driven ceramic-based membranes have proven effective as RO pre-treatments, reducing fouling potential significantly [62]. As shown in Figure 6a, OARO uses a multi-stage process to gradually reduce feed concentration, making final desalination via traditional RO more feasible and less energy-intensive for hypersaline streams [53].

ED is an efficient desalination technology that utilizes an electric field and ion-exchange membranes to selectively transport salt ions from a dilute feed solution into a concentrated brine stream. Electrodialysis has a lower electrical energy consumption of 2.6–5.5 kWh/m^3^ compared to RO for high-salinity applications, with product water TDS ranging from 150 to 500 ppm and water cost of 0.6–1.05 USD/m^3^ [63]. As illustrated in Figure 6b, the process involves applying an electric field across a stack of alternating cation-exchange membranes (CEMs) and anion-exchange membranes (AEMs). These membranes selectively permit the passage of either cations or anions while blocking ions of the opposite charge. ED has been successfully used for desalination of brackish water [64,65,66], as well as in seawater concentration and the treatment of RO brines for salt production [64,67,68]. However, the technology faces several challenges, including decreased current efficiency, unwanted water transport, and membrane resistance. Additionally, osmotic effects cause water to diffuse from the dilute product stream into the concentrated brine, reducing the overall product water recovery [69]. Recent advances in membrane materials have led to the development of highly conductive, anti-fouling AEMs with regenerable antibacterial surfaces, offering improved performance in treating high-salinity wastewater containing organic contaminants [70]. A fundamental comparison between RO and ED indicates that while RO is more energy-efficient for low- to moderate-salinity feeds, ED gains a competitive advantage at high salinities due to its lower operational pressure requirements, which avoid the steep energy demand characteristic of high-pressure RO systems [71].

FO is a desalination technology that uses an osmotic pressure gradient, rather than external pressure as in RO (Figure 6c), to draw water through a membrane from a feed stream into a more concentrated draw solution. The product is a diluted draw solution, which requires a subsequent separation step (e.g., heating [72,73]) to yield pure water. FO offers benefits such as low energy consumption, low scaling potential, and simple pretreatment [74,75]. Its main drawbacks include the high cost and limited availability of suitable draw solutes [76,77] and a typically low water recovery rate. To improve recovery, hybrid FO systems are being developed, which combine FO with processes like membrane distillation to boost efficiency and reduce wastewater [78,79].

MD is a thermally driven separation process that utilizes a hydrophobic membrane to treat high-salinity water, as illustrated in Figure 6d. Driven by a vapor pressure difference, which is typically induced by a temperature gradient, water vapor molecules pass through the porous membrane and condense on the cooler distillate side, yielding high-purity freshwater. A key advantage of MD is its ability to operate at moderate temperatures, allowing the use of low-grade thermal energy sources such as industrial waste heat or solar thermal collectors [80]. Additionally, MD can achieve exceptionally high salt rejection [81], often producing distillate with TDS below 20 ppm. Experimental studies have demonstrated its capability to continuously treat hypersaline brines with TDS concentrations as high as 70,000 mg/L, yielding high-quality product water [82]. To mitigate energy consumption, MD systems can be integrated with renewable or waste heat sources, such as solar or geothermal energy [83,84]. Recent material innovations have significantly improved MD performance in hypersaline environments. For instance, fluorinated covalent organic framework membranes [85] and ultrathin nanocomposite membranes [86] exhibit superior wetting resistance, maintaining stability during long-term operation. Furthermore, MXene-based membranes demonstrate dual functionality: their tailored microstructures ensure high salt rejection [87], while their photothermal properties enable efficient solar-driven evaporation, reducing dependence on external heating [88]. These advancements collectively address critical limitations in MD, including scaling propensity and energy intensity.

#### 3.1.4. Comparison of Treatment Technologies for High-Salinity Water

**Figure 7:**
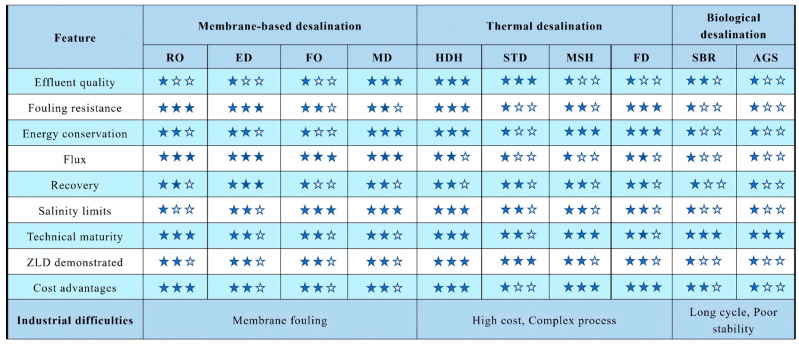
Systematic comparison of different technologies for high-salt wastewater. (The more solid stars there are, the better the performance of this technology in this dimension. Conversely, it performs relatively weakly).

Figure 7 presents a systematic overview and comparison of various high-salt wastewater treatment technologies. Biological methods, such as the sequencing batch reactor (SBR), are well-established and energy-efficient; however, their performance is often limited under high-salinity conditions due to the inhibited activity and survival of microorganisms [15,18]. In contrast, membrane-based desalination technologies produce high-quality effluent but are prone to scaling and high energy consumption [52,54]. Moreover, membrane stability and antifouling performance under high salinity remain major challenges [11].

Different membrane-based processes exhibit distinct characteristics. For example, FO experiences less scaling than reverse osmosis (RO), and the absence of hydraulic pressure in FO may reduce cake layer formation on the membrane surface [90]. Additionally, since FO does not require external pressure to reject contaminants, its operational energy demand and cost are lower [91]. Hybrid membrane systems, such as RO combined with ED, can reduce energy consumption per unit of water produced by 33–36% [92]. Despite its potential in treating saline wastewater, MD suffers from low permeate flux compared to RO, mainly due to temperature polarization [93]. ED shows promise in recovering organic/inorganic substances, heavy metals, and nutrients through selective monovalent ion separation [94]. It also demonstrates lower energy use and operational cost than RO in low-salinity brine treatment [71].

Thermal desalination technologies achieve ZLD but generally exhibit lower efficiency and higher costs compared to membrane-based methods. They are advantageous in specific scenarios, such as treating high-salinity brines or when integrated with renewable energy. Addressing heat exchanger scaling is crucial to improving heat transfer efficiency and expanding MSF’s application to high-salinity wastewater [9].

From an environmental standpoint, membrane technologies incur carbon emissions from chemical cleaning and energy consumption, whereas thermal desalination can achieve a lower carbon footprint when powered by renewable energy [95].

### 3.2. Membrane Scaling Issue

Scaling remains a major challenge in membrane-based treatment of high-salinity wastewater. Such wastewater contains various inorganic salt ions, and differences in the composition and concentration of anions and cations between water sources can lead to chemical reactions that produce crystalline precipitates. This results in incompatibility or poor compatibility between water bodies, ultimately causing scaling. Based on the type of deposits, scaling can be categorized as inorganic, organic, or biological [96].

During desalination or treatment processes, thermal conditions such as temperature, pressure, and pH can disrupt the ionic equilibrium in high-salinity wastewater. This leads to supersaturation of scaling constituents, which subsequently precipitate from the solution, form crystal nuclei, and grow into scale deposits through accumulation and precipitation. The resulting scale can cause pipeline blockage and equipment fouling, compromising system operation. In biological treatment systems, high-salinity conditions can trigger endogenous respiration and cell aggregation in bacteria, promoting the secretion of extracellular polymeric substances (EPS) [97].

For specific inorganic scaling components, quantitative saturation thresholds are critical for guiding targeted scaling control. For gypsum (CaSO_4_·2H_2_O), a dominant inorganic scale in high-salinity wastewater (e.g., flue gas desulfurization wastewater, FGDW), the saturation index (SI) of the bulk solution is a key quantitative indicator. Notably, gypsum crystallization can occur at the membrane interface when the bulk solution SI is still below the critical value, due to interfacial supersaturation induced by temperature and concentration polarization, and stable MD operation for 80 h was achieved by limiting water recovery per cycle to maintain CaSO_4_ concentration below its bulk saturation threshold (SI < 0) [98].

Current approaches to controlling scaling in high-salinity wastewater include chemical and physical scale inhibition methods. Chemical scale inhibition often involves acid cleaning, which can cause corrosion damage to pipelines and equipment. In contrast, a variety of physical scale prevention techniques have been developed, such as high-intensity acoustic shock wave treatment, permanent magnetic descaling, and methods based on crystallization kinetics in aqueous systems [9].

### 3.3. Zero Liquid Discharge

ZLD is an industrial wastewater treatment process designed to completely eliminate liquid waste discharge from a facility [99]. In a ZLD system, all generated wastewater streams are treated and purified to a quality suitable for onsite reuse, while the remaining solid residues or salts are either safely disposed of or recovered for further utilization. The ultimate objective of ZLD is to achieve 100% water recovery within the facility, thereby removing the need for any liquid effluent release into the environment [100]. ZLD is increasingly recognized as a sustainable approach for industrial wastewater management, particularly in sectors such as power generation, chemical manufacturing, and mining, which produce large volumes of highly concentrated wastewater [13,101,102]. Recent applications have expanded to the treatment of produced water in the oil and gas industry using integrated membrane systems [103]. By adopting ZLD, industries can enhance regulatory compliance, reduce freshwater consumption and operational costs, and minimize their environmental footprint. Stricter wastewater disposal regulations remain the primary driver for ZLD adoption.

A crucial stage in the ZLD process is desalination, which involves recovering water from saline waste streams. Desalination can be accomplished through membrane-based technologies—such as RO and ED—or thermal processes, including MED and MSF [99]. RO operates by applying hydraulic pressure exceeding the osmotic pressure of the feed solution to drive water through a semipermeable membrane, thereby retaining most solutes. Hybrid systems combining membrane and thermal technologies are frequently employed to achieve ZLD for high-salinity wastewaters [104]. Advanced membrane processes like bipolar membrane ED have been optimized to produce acids and bases during ZLD treatment of high-salinity brines, enabling concurrent resource recovery and water purification [105]. Meanwhile, innovations in thermal process materials include Hofmeister-effect-based 3D hydrogel sponges that enable non-contact localized crystallization, and bio-inspired solar evaporators that mimic mangrove salt-secretion mechanisms to mitigate salt accumulation [106].

**Figure 8:**
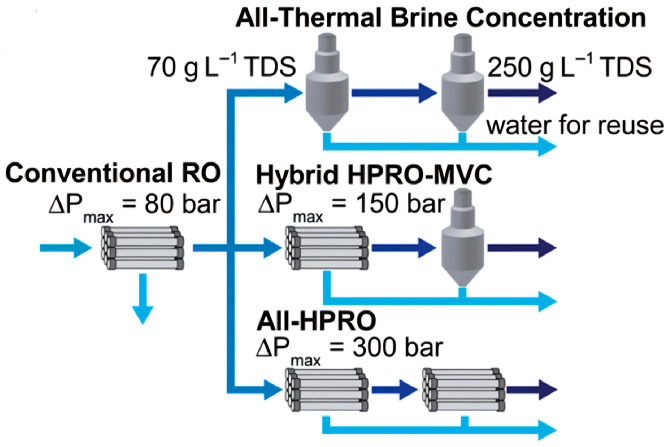
Brine desalination process schematics. Three different process configurations are shown for the concentration of a 70 g/L NaCl solution to 250 g/L.

Owing to its superior energy efficiency, RO has largely supplanted thermal desalination in recent decades for seawater and brackish water desalination aimed at potable water production. Consequently, RO membranes and systems have been optimized for feedwater salinities up to that of seawater. The maximum operating pressure for typical RO systems is around 80 bar, which is sufficient to overcome the retentate osmotic pressure at approximately 50% water recovery [104]. However, due to hydraulic pressure limitations, hypersaline brines are predominantly treated using thermal desalination technologies [99]. These thermal processes are energy- and cost-intensive, which often restricts their implementation in brine desalination prior to final disposal. To enable a direct comparison between membrane and thermal desalination (see Figure 8), the energy requirements of each process can be quantitatively analyzed. Recent advances in material science have aimed at mitigating energy-related challenges, such as gradient graphene spiral sponges that improve solar energy utilization and promote directional salt crystallization for efficient ZLD [36].

Despite its benefits, ZLD implementation can be complex and costly, and its feasibility is influenced by factors such as wastewater characteristics, volumetric load, available treatment technologies, and the prevailing economic and regulatory context [99]. The typical components of ZLD systems are pretreatment, concentrators, and crystallization [5]. Regarding this, some research has been proposed that the end-products of the ZLD system can be sold. Mixed solid salt (>90% sodium chloride) can be used for de-icing roadways, industrial applications, etc. [107]. Moreover, ZLD is energy-intensive and contributes significantly to greenhouse gas (GHG) emissions. Certain pretreatment methods—for instance, acidification followed by degasification—release CO_2_ from the feedwater into the atmosphere. Similarly, using ED to concentrate RO brine increases CO_2_ emissions through both energy consumption and the decarbonation step used for scaling control [108]. When compared to minimal liquid discharge (MLD) systems, which achieve 84.6% water recovery at an energy consumption of 5.4 kWh/m^3^, ZLD systems can attain up to 98.15% recovery but require approximately 10.43 kWh/m^3^ [109]. Degasification and acidification are some of the processes which create air pollution due to the emission of carbon dioxide in the air. Such pre-treatments should be avoided or replaced by other methods [110]. Many other treatment processes like decarbonization, electrodialysis, etc., may lead to an increase in greenhouse gas emissions. Xevgenos et al. [111] observed that emerging membrane technologies consuming lower energy and eco-friendly means of energy need to be encouraged to prevent greenhouse gas emissions. All these strategies can enable the upgradation to ZLD in the future. Emerging alternatives include chemical-free integrated membrane systems that avoid environmental impacts associated with pretreatment chemicals [103]. Brine management remains a challenge, as evidenced by the need for denitrification to treat specific contaminants in reject brine [112]. Nevertheless, growing global water scarcity underscores the value of ZLD in maximizing wastewater recovery. Rising public environmental awareness also drives adoption, as ZLD mitigates the ecological impacts of wastewater discharge and alleviates associated public concerns.

## 4. Challenge and Outlook

**Figure 9:**
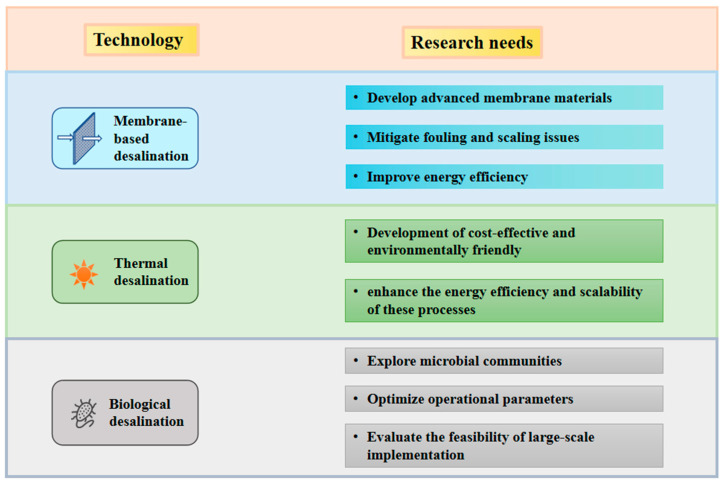
Summary of research needs corresponding to technology.

The extensive body of literature reviewed in this paper demonstrates that considerable and in-depth research has been conducted in the field of high-salinity wastewater treatment. Identifying future research directions for the three major treatment technologies—membrane-based, thermal, and biological processes—is crucial for enhancing treatment efficiency and practicality. As summarized in Figure 9, several key challenges remain in the treatment of high-salinity wastewater.

In membrane technology, the core lies in the development of advanced membrane materials. Fabricating membranes that exhibit high flux, high rejection rates, strong antifouling properties, robust mechanical strength, and low cost is essential for widespread industrial adoption. Thermal concentration processes, while capable of achieving complete desalination, face issues such as scaling that compromise system stability. Moreover, the extended treatment process, high energy consumption, and substantial construction and maintenance costs often render these systems economically challenging. Biological treatment presents an economically viable alternative for high-salinity wastewater, with current research focusing on the cultivation and acclimation of halotolerant microorganisms, as well as the optimization of various bioreactor configurations. The application of halophilic bacteria has shown promise in enhancing the performance of both aerobic and anaerobic biological systems.

Furthermore, the implementation of ZLD technologies requires continued research to improve their efficacy, cost-effectiveness, and environmental sustainability. This includes advancing treatment processes, exploring resource recovery opportunities, and addressing challenges related to the management of concentrated brines. Adopting appropriate treatment strategies—particularly those enabling zero liquid discharge and water reuse—is of great importance for sustainable wastewater management.

It is essential to define clear and targeted research priorities to accelerate the development and industrial application of high-salinity wastewater treatment technologies.

### 4.1. Key Functional Material Innovation

MXene materials, characterized by tunable interlayer spacing and high electrical conductivity, demonstrate considerable potential for improving ion separation efficiency in high-salinity wastewater treatment. In the case of covalent organic frameworks (COFs), molecular design strategies—such as hydrophilic modification and nanochannel optimization—enable a synergistic “pore–charge–wettability” effect, significantly enhancing membrane permeability and ion selectivity in desalination applications. Biomass-derived materials also offer promising alternatives: for instance, fully lignocellulosic bilayer porous hydrogels leverage lignin’s photothermal conversion capability and hierarchical porous structure to achieve efficient solar-driven steam generation, presenting a sustainable pathway for thermal desalination.

### 4.2. Emerging Technologies and Adsorption Desalination

Recent progress in adsorption desalination has broadened the material options available for high-salinity wastewater treatment. Novel adsorbents, including metal–organic frameworks (MOFs) and modified biomass materials, exhibit improved salt adsorption capacity and cycling stability. Among emerging technical routes, dialysis represents a promising approach for treating high-salinity organic wastewater. Operating via a pressure-free, diffusion-driven mechanism with commercial ultrafiltration membranes, this process achieves effective separation of salts and organics while showing superior antifouling performance compared to conventional methods.

### 4.3. Multi-Technology Integration

Integrated processes should aim to combine the complementary strengths of individual technologies. For example, adsorption–membrane hybrid systems can utilize the high salt uptake capacity of advanced adsorbents to pretreat high-salinity wastewater. This step reduces salt content and organic foulants, thereby mitigating membrane fouling and extending membrane lifespan. Similarly, membrane–solar thermal coupled systems can incorporate lignocellulosic bilayer porous hydrogels for solar steam generation. In such configurations, membrane separation first removes macromolecular impurities, after which solar–thermal evaporation enables efficient desalination, lowering dependence on fossil fuel–based thermal units.

### 4.4. Sustainability Enhancement and Industrial Application Prospects

Sustainability improvements should be driven by technological innovation. For instance, integrating renewable energy sources—such as solar-driven steam generation using biomass-derived hydrogels—can replace energy-intensive thermal concentration equipment, supporting progress toward carbon neutrality goals. Salt resource recovery can be coupled with adsorbent regeneration in adsorption desalination systems, allowing high-purity salts to be harvested during the regeneration process. This integration facilitates a “wastewater-to-resource” paradigm. Moreover, the low energy demand and strong antifouling performance of dialysis technology help reduce the carbon footprint and operational costs of treatment systems, positioning it favorably for industrial applications in sectors such as chemical manufacturing and food processing.

## Data Availability

No new data were created or analyzed in this study. Data sharing is not applicable to this article.
